# ﻿*Chaomyia*, a new monotypic genus of Tachininae from the Qinghai-Tibet Plateau, China (Arthropoda, Insecta, Diptera, Tachinidae)

**DOI:** 10.3897/zookeys.1236.141122

**Published:** 2025-05-05

**Authors:** Xusheng Liu, Jiayi Ji, Youpeng Lai, Dong Zhang, Pierfilippo Cerretti, Chuntian Zhang

**Affiliations:** 1 Liaoning Key Laboratory of Biological Evolution and Biodiversity, College of Life Science, Shenyang Normal University, Shenyang 110034, China Shenyang Normal University Shenyang China; 2 Institute of Plant Protection, Academy of Agriculture and Forestry Sciences, Qinghai University, Xining 810016, China Qinghai University Xining China; 3 School of Ecology and Nature Conservation, Beijing Forestry University, Beijing 100083, China Beijing Forestry University Beijing China; 4 Department of Biology and Biotechnology ‘Charles Darwin’, University of Rome ‘Sapienza’, Rome, Italy University of Rome ‘Sapienza’ Roma Italy

**Keywords:** *Chaomyia*, new taxa, Palaearctic, systematics, tachinids

## Abstract

We erected a new genus of Tachinidae in the subfamily Tachininae, *Chaomyia***gen. nov.** for the new species *C.qinghaiensis***sp. nov.** from grasslands of Haiyan County, Qinghai Province, Qinghai-Tibet Plateau, China. *Chaomyia***gen. nov.** is distinguishable from all other genera of Tachininae (Diptera: Tachinidae) based on morphological evidence. Key morphological characters include: small size (ca. 4–5 mm); eye nearly bare; lower facial margin strongly protruding forward in front of vibrissal angle; occiput with only black setulae; lower occiput bulging; bare prosternum; two postpronotal setae; 2 presutural and 3 postsutural dorsocentral setae; 3 pairs of strong marginal scutellar setae, apical setae strong and crossed; wing membrane around crossveins r-m and dm-cu darkened; male fore claws and pulvilli shorter than 5^th^ tarsomere; and mid-dorsal depression of abdominal syntergite 1+2 not reaching hind margin of syntergite, sternites well exposed. A possible affiliation with the tribes Polideini or Ernestiini has been discussed using morphological evidence.

## ﻿Introduction

Despite previous research efforts (O’Hara et al. 2009), the tachinid fauna of China remains significantly understudied (1109 species, 257 genera), especially in the more remote and inaccessible regions of the country, such as the Himalayas and the tropical forests of southern China.

Tachinidae (Diptera) are an important group of insect parasitoids (Stireman et al. 2006); most of them attack lepidopteran larvae, including many pest species (Tschorsnig 2017). In recent years, several initiatives have been undertaken to study the diversity of this group in China. These include expeditions (Chao and Zhou 1996), species cataloguing (O’Hara et al. 2020), and sampling with Malaise traps in China (e.g., Pei et al. 2021; 2024a). The primary aim of these studies is to address ecological questions, such as whether and how parasitoid communities respond to environmental variables (Pei et al. 2024b).

In this context, a Malaise trap was placed in 2023 on the Qinghai-Tibet Plateau at an altitude of 3178 m a.s.l., a region that has been little explored. The Qinghai Province of China is located in the northeastern part of the Qinghai-Tibet Plateau, from the Hoh Xil region in the west (Chao and Zhou 1996) to the Aemye Ma-chhen Range in the east (about 1200 km), and from the southern edge of the Tanggula Mountains in the south to the Kunlun and northern Qilian Mountains in the north (800 km), with the average elevation of Qinghai being over 3000 m a.s.l. The collected material is currently being studied in detail using morphological techniques. As part of our results, we have found nine specimens of an unidentified species that share several characters with members of the tribe Polideini and Ernestiini of Tachininae but apparently cannot be assigned to any known genera. The aim of this study is to describe the new species, establish a new monotypic genus for it and provide explicit arguments for the placement of this new taxon within the tribe Polideini sensu O’Hara, 2002 or Ernestiini Townsend, 1912.

## ﻿Material and methods

### ﻿Taxon sampling and terminology

**Figure 1.**
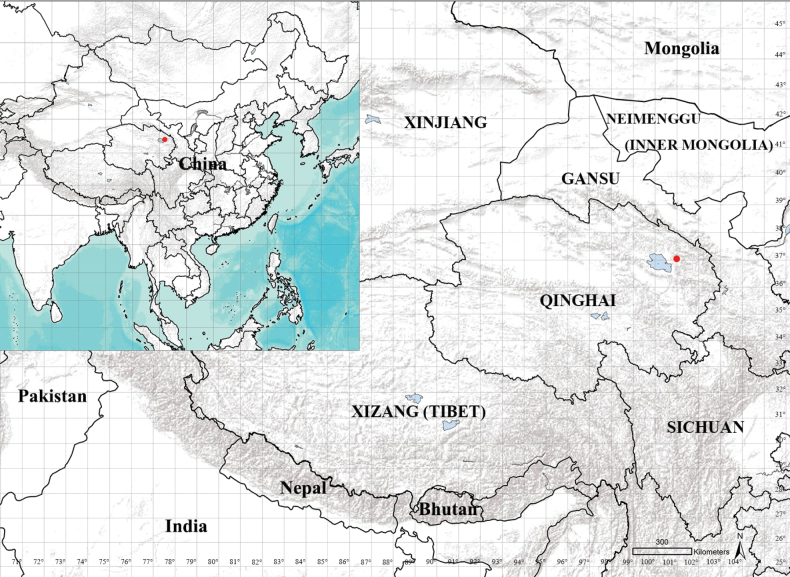
Type locality (Halejing, 3178.7 m, Haiyan County, Haibei Prefecture) of *Chaomyiaqinghaiensis* sp. nov. from Qinghai, China.

The specimens were collected in northern Qinghai, on the Qinghai-Tibet Plateau, China (Fig. 1). Specifically, the collection site was Halejing Mongolian Village (37°2'3"N, 100°59'52"E; elevation: 3178.7 m), located in Haiyan County, Haibei Tibetan Autonomous Prefecture. The sampling was conducted on September 5, 2023, using a Malaise trap by Jiayi Ji. Qinghai Province spans an area of 721,200 km^2^ and is situated between 89°35'–103°04'E and 31°40'–39°19'N.

Morphological terminology and measurements used follow Tschorsnig and Richter (1998) and Cumming and Wood (2017). Preliminary identifications were made using the key of Tschorsnig and Richter (1998) and the matrix-based interactive key of Cerretti et al. (2012).

Specimens were examined using a Leica M205 C stereomicroscope. Digital images of the heads, bodies of male and female were taken with a Canon EOS 60D camera and the images were combined using Helicon Focus v. 8.1.0. Dissections of male and female terminalia were carried out following the method described by O’Hara (2002), digital images were taken with Leica M205 A stereomicroscope and images were combined using Leica Application Suiter v. 4.12.0. Dissected terminalia were placed in glycerin in a small plastic tube and pinned together with the source specimen. The species distribution map was generated with ArcGIS v. 10.2 (ESRI Inc.). The tachinid specimens of this study were deposited in the Insect Collection of Shenyang Normal University (**SYNU**), Shenyang and the Institute of Plant Protection, Academy of Agriculture and Forestry Sciences, Qinghai University (**QHU**), Xining, China.

## ﻿Systematics

**Subfamily Tachininae Rondani, 1859**

**Tribe Polideini sensu O’Hara, 2002 or Ernestiini Townsend, 1912**

### ﻿ Chaomyia

Taxon classificationAnimaliaDipteraTachinidae

C. Zhang & Cerretti gen. nov.

30639744-4486-5C35-9FC0-A8C9EEDA3517

https://zoobank.org/FEC2D23C-9C66-4491-84E9-F48DA6D8B414

Figs 2 , 3 , 4

#### Type species.

*Chaomyiaqinghaiensis* C. Zhang & Cerretti, sp. nov., by present designation.

#### Remarks.

Tribe Affiliation. *Chaomyiaqinghaiensis* gen. & sp. nov. exhibits several characteristic morphological features, including a strongly protruding lower facial margin, which is well visible in front of vibrissal angle, a bare prosternum, and preapical anterodorsal seta of the fore tibia only slightly shorter than the preapical dorsal seta. Moreover, its male terminalia display a tergite 6 that is not longitudinally divided into hemitergites, a weakly sclerotized lateroventral area of distiphallus, and basiphallus that runs nearly parallel to distiphallus. This combination of likely derived character states is consistent with the assignment of the genus to the subfamily Tachininae (Tschorsnig and Herting 1994; Cerretti et al. 2014).

Recent phylogenetic reconstructions based on molecular data support Tachininae as a monophyletic group, with the exclusion of Macquartini and Myiophasiini, a clade of parasitoids of beetle larvae that is widely distributed globally (Stireman et al. 2019). Adult Macquartiini and Myiophasiini share having males with strongly narrow frons, a body ground color which is mostly black and, although very variable, a male phallus lacking a sclerotize medioventral sclerite, and cerci not fused into a syncercus. *Chaomyia* has male cerci fused into a syncercus, membranous posterior sclerite of distiphallus, well-developed anepimeral seta, male hypandial arms that are posteromedially fused and completely encircle the base of the phallus, male epandrium with setae dorsally, and a distinctly shaped medioventral sclerite of the distiphallus (see O’Hara 2002, who described this as the anterior sclerite of the distiphallus). For these reasons, we think *Chaomyia* cannot be assigned to either of these two tribes.

*Chaomyia* shares most of the putative synapomorphies identified by O’Hara (2002) supporting the monophyly of the tachinine tribe Polideini. However, it lacks certain diagnostic features: the male surstylus in *Chaomyia* is nearly bare apically, rather than bearing spine-like setae; male sternite 5 possesses micropubescence along the inner margin of the posteromedian cleft, rather than distinct microspines; and the posterior thoracic spiracle is not uniformly haired on both sides but instead is covered by flap-like setulae originating only from its posterior margin. We interpret these morphological differences as evidence that *Chaomyia* could represent a member of the Polideini, pending confirmation of its phylogenetic position through molecular data.

In the key to Palaearctic genera by Tschorsnig and Richter (1998: 756, 772, 777), our specimens key out to *Synactia* Villeneuve of the tribe Ernestiini. However, *Synactia* differs from *Chaomyia* in several respects, including: densely hairy eyes, facial ridge with setae on lower 2/5 to 2/3, lower facial margin not protruding forward, arista thickened on basal 1/2 or more, R_4+5_ with setulae at the base, and median excavation of syntergite 1+2 reaching to hind margin. Given these differences, *Chaomyia* cannot be confidently placed within *Synactia* or most other members of Ernestiini. Nevertheless, the available morphological evidence provisionally supports the assignment of *Chaomyia* to either the tribe Polideini or Ernestiini, pending further study.

#### Evidence supporting *Chaomyia* as new genus.

Following a thorough examination and comparison of *C.qinghaiensis* sp. nov. with both collection material and scientific literature (Crosskey 1976; Wood 1987; Tschorsnig and Richter 1998; Wood and Zumbado 2010; Cerretti et al. 2012), we conclude that it exhibits a unique combination of morphological traits among Tachininae. This combination of characters is considered apomorphic, providing a strong justification for the establishment of a new genus to accommodate the species from Halejing.

#### Description.

**Male and female.** Small-sized tachinid flies.

**Figure 2.**
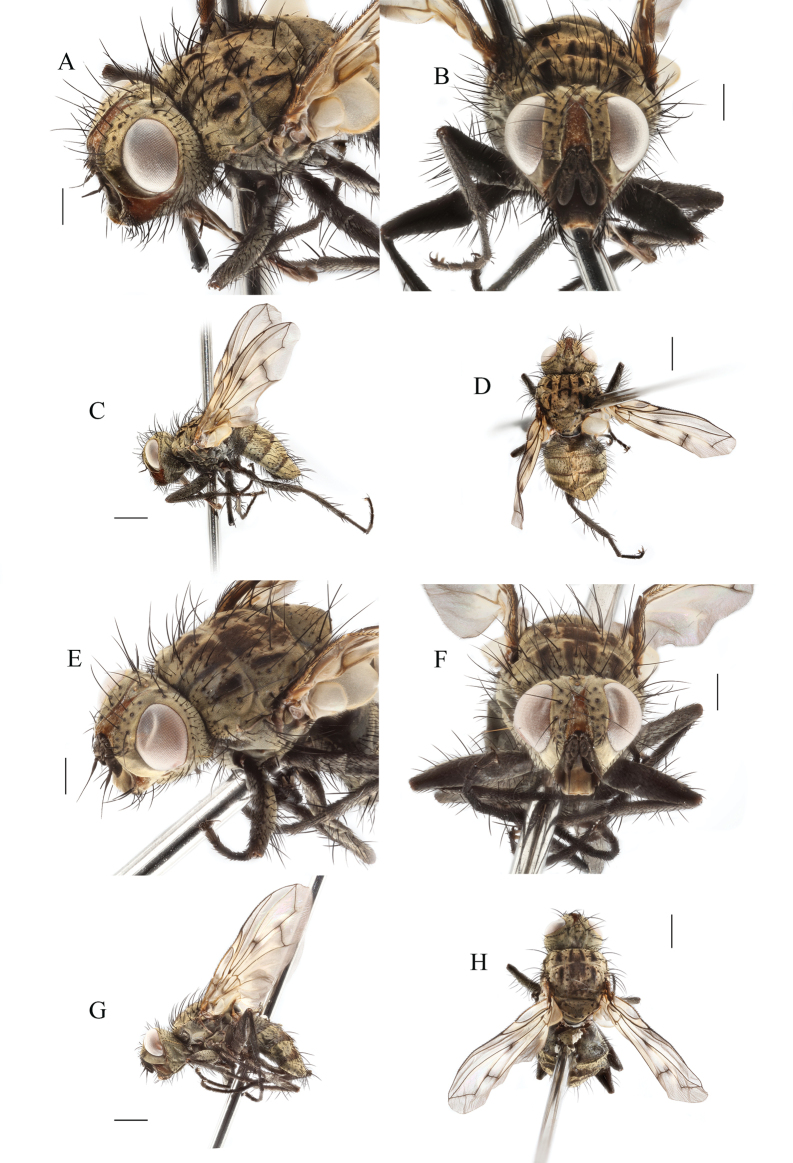
*Chaomyiaqinghaiensis* sp. nov. **A–D**, ♂; **E–H**, ♀. **A, E, C, G** head and body in lateral view **B, F** head in anterior view **D, H** body in dorsal views. Scale bars 0.5 mm (**A, B, E, F**); 1.0 mm (**C**, **D**, **G**, **H**).

***Head*** (Fig. 2A, B, E, F). Eyes nearly bare, with sparse very short ommatrichia. Frons 1.26–1.41 (male) and 1.33–1.60 (female) of eye width. Frontal vitta slightly narrower than fronto-orbital plate in front of ocellar triangle. Parafacial bare, at most as wide as postpedicel in anterior view. Face without facial carina; lower margin of face distinctly protruding forward in front of vibrissal angle and visible in lateral view. Gena 0.5–0.6 of eye height. Fronto-orbital plate with 5–7 inclinate frontal setae; 4–5 outer orbital setae, anterior one or two proclinate, posterior two or three lateroclinate in male; 4 outer orbital setae, anterior one proclinate and posterior three lateroclinate in female. Occiput with only black hairs behind postocular row, ventral part bulging. Arista bare, thickened at basal 1/3 to 2/5. Palpus about 3/4 as long as postpedicel, with 2–3 apical setae. Prementum 7–8 times as long as wide. Labellum small.

***Thorax*** (Fig. 2C, D, G, H). Body densely covered with yellowish pruinosity. Prosternum bare. Postpronotal lobe with two setae and some hairs; first postsutural supra-alar seta about as long as the first postsutural intra-alar seta; one anepimeral seta extending to posterior margin of upper calypter. Scutellum entirely black, with 3 pairs of marginal setae, subapical scutellar seta not extending back to apices of strong crossed apical scutellar setae. Spiracles dark and small; posterior spiracle posterior fringe visibly larger than anterior fringe, proepimeral setae directed upward. Base of vein R_4+5_ with 2–4 small setulae dorsally and ventrally.

***Wing*** membrane around crossveins r-m and dm-cu darkened. Vein M evenly bent, bluntly angled, extending to wing margin making cell r_4+5_ closed at wing margin or merged to vein R_4+5_ to form a very short petiole.

***Leg.*** Fore claws and pulvilli shorter than 5^th^ tarsomere. Fore tibia with 2–3 short anterodorsal, 2 posterior and 1 posterodorsal setae, preapical anterodorsal seta approximately as long as preapical dorsal seta or just slightly shorter. Hind tibia with 2 preapical dorsal setae, preapical anteroventral seta about as long as preapical posteroventral seta.

***Abdomen*.** Mid-dorsal depression on abdominal syntergite 1+2 not reaching to hind margin. Syntergites 1+2 to tergite 4 each with dark transverse band only on lateral posterior 1/4–1/5. Sternite 1 hairy; the other sternites exposed.

**Figure 3.**
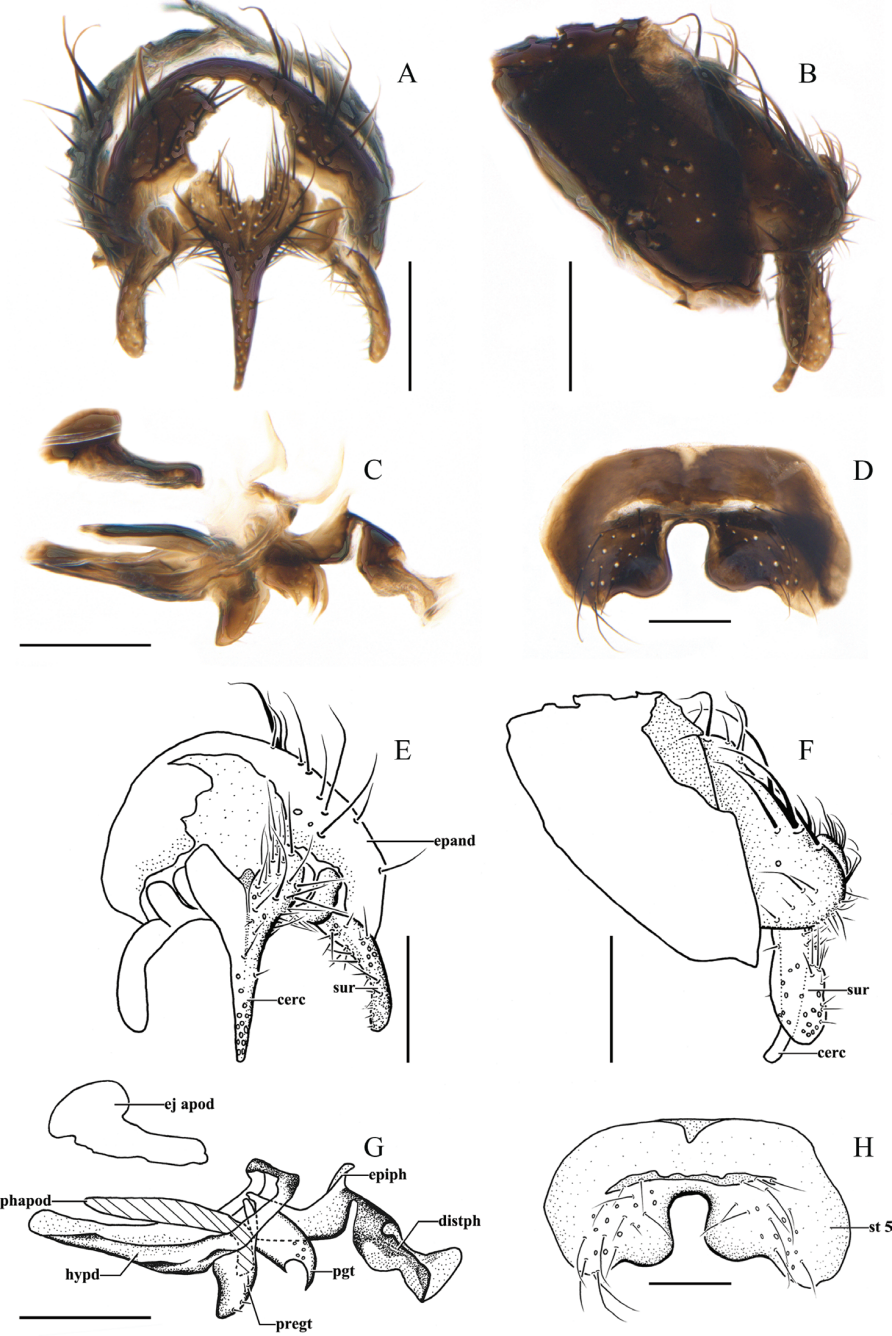
*Chaomyiaqinghaiensis* sp. nov. **A–H**, ♂, **A, E; B, F** cerci, surstyli and epandrium of male in caudal and lateral views **C, G** phallus (aedeagal apodeme, ejaculatory apodeme, hypandrium, epiphallus, pregonite, postgonite, basiphallus and distiphallus) of male in lateral view **D, H** sternite 5 in ventral view. Scale bars: 0.1 mm.

***Male terminalia*** (Fig. 3A–H). Tergite 6 not divided medially into two hemitergites. In ventral view, sternite 5 nearly square with a deep, U-shaped median cleft which is approximately 1/2 length of the sternite; lateral lobe of sternite 5 with an inner protrusion bluntly rounded apically. In caudal view, cerci fused to form a syncercus, narrowed on apical 2/3 and pointed, not separated apically; surstylus narrowed and bent inwardly and blunt apically. In lateral view, syncercus longer than surstylus, bent backward apically; surstylus thick, bluntly round apically, without teeth or spines apically. Hypandrium broadly expanded posteriorly on both sides, hypandrial arms narrowly fused dorsally; pregonite broad, lobe-like; postgonite pointed apically; phallus well developed, basiphallus with a weakly sclerotized epiphallus in distal position; medioventral ridge of distiphallus present and well developed; lateroventral region of the distiphallus membranous, ejaculatory apodeme large and process fan-shaped.

**Figure 4.**
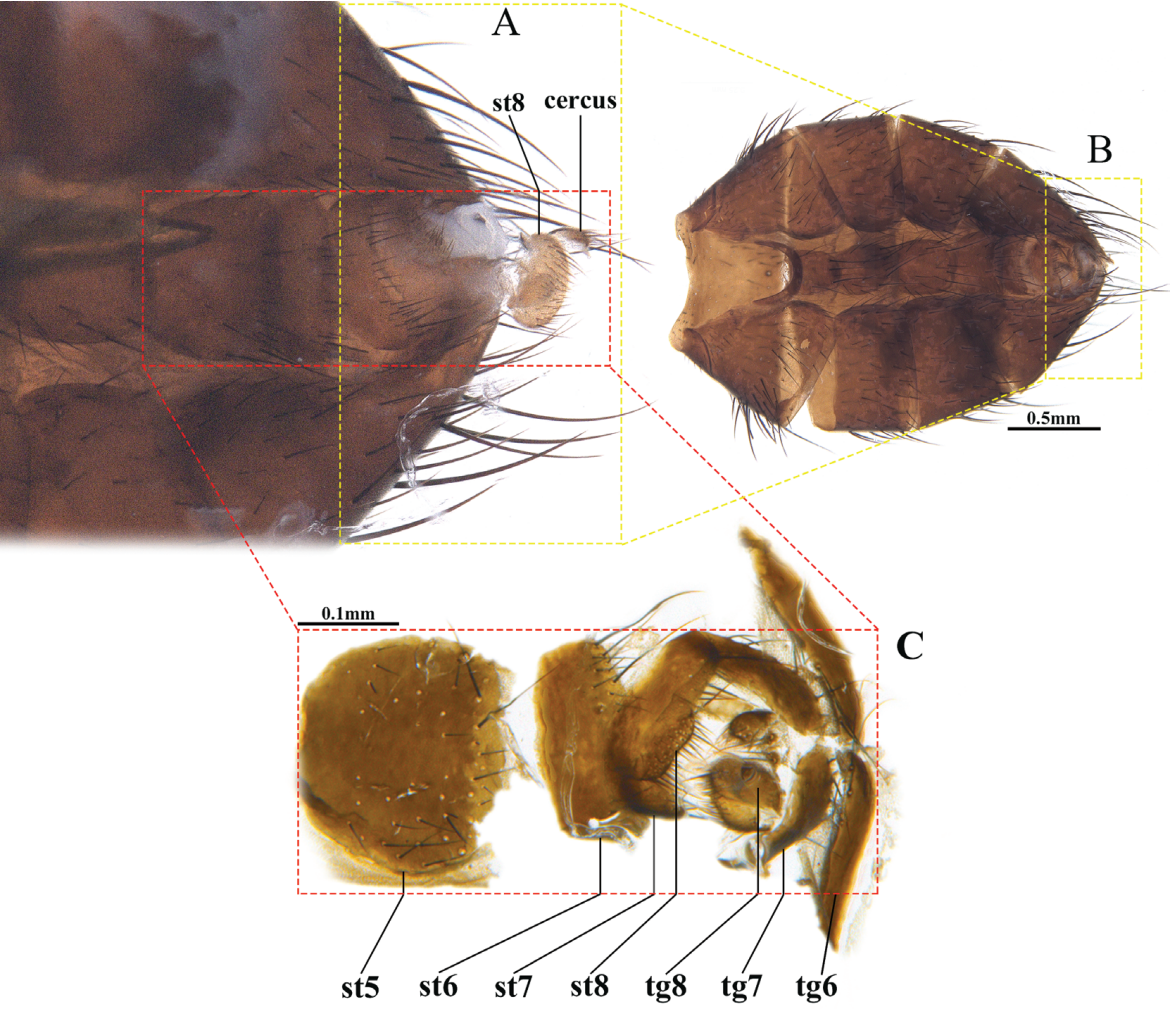
*Chaomyiaqinghaiensis* sp. nov. ♀ Female abdomen in ventral view **A** postabdomen **B** abdomen **C** postabdomen in detail, st = sternite, tg = tergite, sternites 5, 6, 7, 8 and tergites 6, 7, 8.

***Female terminalia*** (Fig. 4). Sternite 5 nearly square, with median and posterior setae; tergites 6–8 not divided into 2 hemiterigites, with a row of strong setulae; sternites 6–8 with posterior setulae.

#### Distribution.

China: Halejing, 3178.7 m, Haiyan County, Haibei Prefecture, Qinghai Province (Fig. 1).

#### Etymology.

The genus name is formed by the name of Chao Cheiming (= Zhao Jianming), a late Chinese dipterologist, plus the Greek *myia*, meaning “fly”, in memory of Chao’s great contributions to Tachinidae taxonomy of China. *Chaomyia* is treated as feminine.

### ﻿ Chaomyia qinghaiensis

Taxon classificationAnimaliaDipteraTachinidae

C. Zhang & Cerretti sp. nov.

59C557A5-2B64-5B9D-A44A-B4B2EB520DF0

https://zoobank.org/F2E04888-2975-4ED7-AFFA-D9D639849793

Figs 2 , 3 , 4

#### Material examined.

***Holotype***: China • ♂ (SYNU-QH 230001); Qinghai Province; Haiyan County, Haibei Tibetan Autonomous Prefecture, Halejing Mongolian Village; 37.0342°N, 100.9977°E; 3178.7 m elev., 5.IX.2023; Malaise trap-Jiayi Ji (SYNU). ***Paratypes***: • 4 ♂ 4 ♀ (SYNU-QH 230002-9), same data as holotype (Fig. 1).

#### Diagnosis.

Eyes nearly bare. Head and body densely covered with yellowish pruinosity. Frons more than eye width in both sexes, fronto-orbital plate of male with 5 to 7 inclinate frontal setae, 1~2 proclinate outer orbital setae and 2~3 lateroclinate outer orbital setae, gena height 0.5–0.6 of eye height, lower facial margin distinctly protruding forward, occiput with only black setulae. Wing with dark clouded at crossveins r-m and dm-cu. Male fore claws and pulvilli shorter than 5^th^ tarsomere. Mid-dorsal depression of abdominal syntergite 1+2 extending on basal half, sternites well exposed.

#### Description.

**Male.** Body length 4.5–5.2 mm.

***Head*** (Fig. 2A, B). Eyes nearly bare, with only sparsely whitish short ommatrichia. Frontal vitta reddish brown. Frontal-orbital plate and parafacial covered with yellow pruinosity. Gena reddish brown. Antenna and arista black. Palpus dark brown. Prementum gleaming black. Frons 1.26–1.41 times of eye width. Frontal vitta slightly narrower than fronto-orbital plate in front of ocellar triangle. Parafacial bare, at most as wide as postpedicel in frontal view, as wide as postpedicel in lateral view. Median facial carina absent, lower facial margin distinctly protruding forward in front of vibrissal angle. Gena height about as long as postpedicel, 0.5–0.6 of eye height; genal dilation well developed. Fronto-orbital plate wide, with 2 rows of setae, 5 to 7 inclinate frontal setae, reaching on parafacial, the middle of pedicel; 1–2 proclinate outer orbital setae and 2–3 lateroclinate outer orbital setae; 2 ocellar setae situated between the anterior and posterior ocelli, proclinate, well developed, nearly as long as frontal setae close to ocellar triangle. Inner vertical seta about 0.7 of eye height; outer vertical seta 2/3–3/4 as long as inner vertical seta. Facial ridge more or less straight, with more or less erect short setae on lower 1/3. Vibrissa inserted level with lower facial margin or below, slightly longer than subvibrissal setae; subvibrissal ridge with 4–5 strong subvibrissal setae mixed with some fine setulae. Postpedicel 2.0–2.3 times as long as pedicel; pedicel with 1 long seta on dorsal surface, which is slightly longer than pedicel. Arista thickened on basal 1/3 to 2/5, about as long as antenna, 2^nd^ aristomere 2.0–2.5 times as long as wide. Palpus filiform, not apically enlarged, about 3/4 as long as postpedicel, with 2–3 apical setae. Prementum 7–8 times as long as its diameter. Labellum small.

***Thorax*** (Fig. 2C, D). Scutum covered with yellow pruinosity and four dark longitudinal vittae on dorsum, presutural inner vitta about 1/4 as wide as pruinose portion between inner and outer vittae, presutural outer vittae approximately triangular; inner vittae only on anterior 1/3 of postsutural scutum. Two presutural and 2 postsutural acrostichal setae; 2 presutural and 3 postsutural dorsocentral setae; 2 postsutural intra-alar setae separated by a distance greater than the distance between the first seta and the suture; first postsutural supra-alar seta slightly longer than notopleural setae, about as long as the first postsutural intra-alar seta. Scutellum with 3 pairs of marginal setae, subapical scutellar seta not extending back to level of apices of strong crossed apical scutellar setae and tilted upwards by 30–60°. Prosternum bare; postpronotal lobe with two setae; proepisternum bare; anepisternum with two antero-upper weak setae and a row of 4–6 posterior setae. Anepimeron with 1 seta extending to anterior 1/2 of lower calypter; katepisternum with 3 setae (2 anterior and 1 posterior, anterior-lower one weaker) with 3 seta-like hairs between anterior and posterior setae. Katepimeron bare. Anatergite below calypter and katatergite bare. Posterior spiracle small and posterior fringe visibly larger than anterior fringe. Postmetacoxal area membranous.

***Wing*** long and narrow, hyaline, brownish. Tegula and basicosta dark brown. Second costal section bare ventrally, costal spine short or absent. Base of R_4+5_ with 2–4 small setulae dorsally and ventrally. Relative lengths of 2^nd^, 3^rd^ and 4^th^ costal sectors approximately 1:4:2. Vein M evenly bent, bluntly angled, extending to wing margin, making cell r_4+5_ closed or short petiolate. Costal sector 4 distinctly longer than costal sector 6. Section of vein M between r-m and dm-cu about as long as section between dm-cu and bend of M. Crossvein dm-cu nearly straight and not exceptionally oblique. Section of vein M between dm-cu and bend at least 2 times distance between the bend and wing posterior margin. Vein CuA_1_ bare. Lower calypters milky white with yellowish, bare on dorsal surface, inner edge of lower calypters close to outer edge of scutellum, its outer margin not strongly convex. Halter brownish yellow, only darker at apex and larger than posterior spiracle.

***Leg*** dark brown. Fore claws and pulvilli shorter than 5^th^ tarsomere. Fore tibia with 2–3 short anterodorsal, 2 posterior and 1 posterodorsal setae, preapical anterodorsal seta slightly shorter or approximately as long as preapical dorsal seta or just slightly shorter. Mid femur with 1 anterior, 1 preapical dorsal seta and a row of posteroventral setae on basal half; mid tibia with 2 anterodorsal, 2–3 posterior and 1 ventral setae. Hind coxa bare on posterodorsal surface; hind femur separately with a row of anteroventral, posteroventral setae and anterodorsal setae, 2 posterodorsal setae on apical 1/3; hind tibia with 3–4 anterodorsal, 3 posterodorsal and 2 ventral setae, 2 preapical dorsal setae, preapical anteroventral seta about as long as preapical posteroventral seta.

***Abdomen*** long ovate, almost covered with grayish-yellow pruinosity, tergites not fused dorsally, syntergite 1+2 to tergite 4 each with dark transverse band on lateral posterior 1/5. Mid-dorsal depression of syntergite 1+2 extending on the proximal half, syntergite 1+2 with 2 median marginal and 1–3 lateral marginal setae. Tergite 3 with 2 median marginal and 2 weak median discal setae, 1 pair of lateral marginal and 2 pairs of lateral discal setae. Tergite 4 with a row of marginal setae, 2 median discal and 2 pairs of lateral discal setae. Tergite 5 inverted trapezoid-like, approximately the same length as tergite 4, with a row of a marginal setae, 2 median discal setae, 2–3 pairs of lateral discal setae. Ventral surface of tergites 4 and 5 covered with thin decumbent hairs on a shiny, non-pruinose cuticle. Sternite 1 hairy, the other sternites exposed; sternites 2 to 4 each with 3–4 setae on posterior portion. Sternite 5 and male terminalia are the same as generic descriptions as shown in Fig. 3A–H.

**Female** (Figs 2E–H; 4). Frons 1.33–1.60 of eye width. Parafacial equal or slightly wider than postpedicel in frontal view, wider than postpedicel in lateral view. Gena higher than antennal length in lateral view. Six pairs of frontal setae. Postpedicel 1.5–2.0 times as long as pedicel. Mid tibia with 4–5 posterodorsal setae. Hind tibia with 2–3 ventral setae. Abdominal syntergite 1+2 without median marginal seta; tergite 5 inverted cone-like. Other features are same as in male.

#### Distribution.

China: Halejing, 3178.7 m, Haiyan County, Haibei Prefecture, Qinghai Province (Fig. 1).

#### Etymology.

The species name is taken from the type locality, Qinghai Province, China. Adjective.

## ﻿Discussion

This study is yet another example, among many now available in the global scientific literature, of how environments increasingly threatened by anthropogenic activities risk erasing the traces of species whose identity and functions within the ecosystem are still unknown (Wilson 2017). Parasitoids are key organisms in the functioning of all terrestrial ecosystems, and we cannot predict the impact of their extinction or population reduction if we do not even know how many species exist. This is particularly true at a time when human-induced climate change will also affect human activities to such an extent that many populations will be forced to migrate (Kaczan and Orgill-Meyer 2020), possibly to higher altitudes. Parasitoid communities regulate the population dynamics of their hosts, which are often primary consumers. Any imbalance in these dynamics could lead to instability, with potentially dramatic consequences for local economies and human health (Di Marco et al. 2023). Describing biodiversity is only the first but essential step, and it is up to taxonomists to accelerate the pace of this description.

The *Chaomyiaqinghaiensis* gen. & sp. nov. specimens were found on a plateau at high altitudes where anthropogenic impact, although still limited, is mainly characterized by pastoralism and some cultivations. This habitat and region have been rarely explored previously and what we found is remarkable. The specimens display a unique combination of morphological features not shared by any other tachinid genus described from the Palaearctic (Tschorsnig and Richter 1998; Cerretti et al. 2012) and Oriental (Crosskey 1976) regions. For this reason, we propose to establish a new genus for them.

## Supplementary Material

XML Treatment for Chaomyia

XML Treatment for Chaomyia qinghaiensis

## ﻿Additional information

### Author ORCIDs

Xusheng Liu https://orcid.org/0009-0001-3758-1465

Jiayi Ji https://orcid.org/0009-0001-7402-8239

Youpeng Lai https://orcid.org/0000-0001-9188-9906

Dong Zhang https://orcid.org/0000-0001-6427-7867

Pierfilippo Cerretti https://orcid.org/0000-0002-9204-3352

Chuntian Zhang https://orcid.org/0000-0002-9514-0502

### Data availability

All of the data that support the findings of this study are available in the main text or Supplementary Information.

## Supplementary materials

Supplementary material 1 Taxa, voucher and GenBank accession numbers used in this study Data type doc https://binary.pensoft.net/file/1322682This dataset is made available under the Open Database License (http://opendatacommons.org/licenses/odbl/1.0/). The Open Database License (ODbL) is a license agreement intended to allow users to freely share, modify, and use this Dataset while maintaining this same freedom for others, provided that the original source and author(s) are credited.Xusheng Liu, Jiayi Ji, Youpeng Lai, Dong Zhang, Pierfilippo Cerretti, Chuntian Zhang
