# Supplementary material for: ﻿Chaomyia, a new monotypic genus of Tachininae from the Qinghai-Tibet Plateau, China (Arthropoda, Insecta, Diptera, Tachinidae)
Source: Zookeys. 2025 May 5;1236:283–95. doi: 10.3897/zookeys.1236.141122 (PMC12070068; doi:10.3897/zookeys.1236.141122)
Supplement: Supplementary material 1 — Taxa, voucher and GenBank accession numbers used in this study [file zookeys-1236-283_article-141122__-s001.doc]

**Table S1** Taxa, voucher and GenBank accession numbers used in this study.

| **Subfamily** | **Tribe** | **Taxa** | **Accession No.** | **Source** |
| --- | --- | --- | --- | --- |
| Tachininae | Ernestiini | *Chrysosomopsis aurata* | KX844227 | Pohjoismaki,J.L. et al., 2016 |
|  |  | *Flavicorniculum planiforceps* | NC086575 | Zhang,P., 2024 |
|  |  | *Gymnocheta viridis* | OY101446 | Unknown, 2023 |
|  |  | *Hyalurgus flavipes* | OQ835465 | Zhang,P., 2023 |
|  |  | *Janthinomyia* sp. | MK644822 | Hou,P., 2020 |
|  |  | *Linnaemya picta* | OP747159 | Li,H., 2023 |
|  |  | *Panzeria anthophila* | OQ835464 | Zhang,P., 2023 |
|  |  | *Zophomyia temula* | MN868806 | Ferreira,S. et al., 2020 |
|  | Germariini | *Germaria angustata* | KX843804 | Pohjoismaki,J.L. et al., 2016 |
|  | Germariochaetini | *Germariochaeta clavata* | KX843829 | Pohjoismaki,J.L. et al., 2016 |
|  | Graphogastrini | *Graphogaster* sp*.* | MF834173 | deWaard,J.R. et al., 2022 |
|  |  | *Phytomyptera* sp*.* | OP599375 | Levesque-Beaudin,V. et al., 2023 |
|  | Leskiini | *Aphria ocypterata* | MF828702 | deWaard,J.R. et al., 2022 |
|  |  | *Bithia* sp*.* | OP650045 | Li,H., 2023 |
|  |  | *Demoticus plebejus* | KX844435 | Pohjoismaki,J.L. et al., 2016 |
|  |  | *Fischeria bicolor* | MN411081 | Dikow,T., 2020 |
|  |  | *Trichoformosomyia* sp. | MN411246 | Dikow,T., 2020 |
|  |  | *Leskia* sp. | JQ574547 | Unknown, 2012 |
|  |  | *Solieria pacifica* | KX844232 | Pohjoismaki,J.L. et al., 2016 |
|  | Macquartiini | *Anthomyiopsis nigrisquamata* | KX843802 | Pohjoismaki,J.L. et al., 2016 |
|  |  | *Macquartia* sp*.* | OL681848 | Li,H., 2022 |
|  | Megaprosopini | *Dexiosoma caninum* | KX843963 | Pohjoismaki,J.L. et al., 2016 |
|  | Minthoini | *Mintho rufiventris* | MN868871 | Ferreira,S. et al., 2020 |
|  | Neaerini | *Neaera atra* | KX844126 | Pohjoismaki,J.L. et al., 2016 |
|  | Nemoraeini | *Nemoraea pellucida* | OP747135 | Li,H., 2023 |
|  | Ormiini | *Aulacephala maculithorax* | MN411137 | Dikow,T., 2020 |
|  |  | *Therobia leonidei* | KX844425 | Pohjoismaki,J.L. et al., 2016 |
|  | Pelatachinini | *Pelatachina tibialis* | KX843706 | Pohjoismaki,J.L. et al., 2016 |
|  | Polideini | *Lydina aenea* | NC063609 | Leerhoei,F., 2023 |
|  |  | *Lypha ruficauda* | KX843702 | Pohjoismaki,J.L. et al., 2016 |
|  | Siphonini | *Actia diffidens* | MG474125 | deWaard, J.R. et al., 2022 |
|  |  | *Siphona* sp*.* | PP232028 | Zhang,P., 2024 |
|  |  | *Ceromya bicolor* | KX844208 | Pohjoismaki,J.L. et al., 2016 |
|  |  | *Peribaea hertingi* | KX844097 | Pohjoismaki,J.L. et al., 2016 |
|  | Tachinini | *Mikia* sp*.* | PP232027 | Zhang,P., 2024 |
|  |  | *Peleteria iavana* | NC063086 | Zhang,P., 2023 |
|  |  | *Tachina sobria* | NC086865 | Zhang,P., 2024 |
| Dexiinae | Dexiini | *Dexia rustica* | MN868902 | Ferreira,S.A., 2020 |
| Exorstinae | Exoristini | *Exorista tubulosa* | OQ611410 | Kilian,I.C., 2024 |
| Phasiinae | Phasiini | *Phasia fenestrate* | MG163622 | Dewaard,J.R., 2018 |
